# 2,4-Dichloro-*N*-(2-chloro­phen­yl)benzene­sulfonamide

**DOI:** 10.1107/S1600536810021306

**Published:** 2010-06-16

**Authors:** B. Thimme Gowda, Sabine Foro, P. G. Nirmala, Hartmut Fuess

**Affiliations:** a Department of Chemistry, Mangalore University, Mangalagangotri 574 199, Mangalore, India; b Institute of Materials Science, Darmstadt University of Technology, Petersenstrasse 23, D-64287 Darmstadt, Germany

## Abstract

In the title compound, C_12_H_8_Cl_3_NO_2_S, the conformation of the N—H bond in the C—SO_2_—NH—C segment is *syn* to the *ortho*-Cl in the aniline ring. The dihedral angle between the two benzene rings is 74.3 (1)°. An intra­molecular N—H⋯Cl hydrogen bond occurs. In the crystal, pairs of N—H⋯O hydrogen bonds link the mol­ecules into dimers.

## Related literature

For the preparation of the title compound, see: Savitha & Gowda (2006 ▶). For our studies of the effect of substituents on the structures of *N*-(ar­yl)aryl­sulfonamides, see: Gowda *et al.* (2010**a* ▶,b* ▶); Nirmala *et al.* (2010 ▶). For related structures, see: Gelbrich *et al.* (2007 ▶); Perlovich *et al.* (2006 ▶).
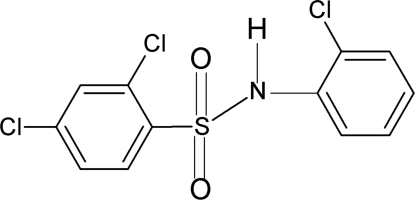


## Experimental

### 

#### Crystal data

C_12_H_8_Cl_3_NO_2_S
*M* *_r_* = 336.60
Orthorhombic, 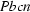

*a* = 10.5639 (8) Å
*b* = 16.279 (1) Å
*c* = 16.693 (1) Å
*V* = 2870.7 (3) Å^3^
*Z* = 8
Mo *K*α radiation
μ = 0.78 mm^−1^
*T* = 299 K
0.30 × 0.24 × 0.22 mm

#### Data collection

Oxford Diffraction Xcalibur diffractometer with a Sapphire CCD detector
Absorption correction: multi-scan (*CrysAlis RED*; Oxford Diffraction, 2009 ▶) *T*_min_ = 0.800, *T*_max_ = 0.847
8054 measured reflections
2938 independent reflections
2054 reflections with *I* > 2σ(*I*)
*R*_int_ = 0.019

#### Refinement

*R*[*F* ^2^ > 2σ(*F* ^2^)] = 0.042
*wR*(*F* ^2^) = 0.107
*S* = 1.01
2938 reflections
175 parameters
1 restraint
H atoms treated by a mixture of independent and constrained refinement
Δρ_max_ = 0.36 e Å^−3^
Δρ_min_ = −0.35 e Å^−3^

### 

Data collection: *CrysAlis CCD* (Oxford Diffraction, 2009 ▶); cell refinement: *CrysAlis RED* (Oxford Diffraction, 2009 ▶); data reduction: *CrysAlis RED*; program(s) used to solve structure: *SHELXS97* (Sheldrick, 2008 ▶); program(s) used to refine structure: *SHELXL97* (Sheldrick, 2008 ▶); molecular graphics: *PLATON* (Spek, 2009 ▶); software used to prepare material for publication: *SHELXL97*.

## Supplementary Material

Crystal structure: contains datablocks I, global. DOI: 10.1107/S1600536810021306/bq2220sup1.cif

Structure factors: contains datablocks I. DOI: 10.1107/S1600536810021306/bq2220Isup2.hkl

Additional supplementary materials:  crystallographic information; 3D view; checkCIF report

## Figures and Tables

**Table 1:** Hydrogen-bond geometry (Å, °)

*D*—H⋯*A*	*D*—H	H⋯*A*	*D*⋯*A*	*D*—H⋯*A*
N1—H1*N*⋯O1^i^	0.86 (1)	2.23 (2)	3.007 (3)	152 (2)
N1—H1*N*⋯Cl3	0.86 (1)	2.57 (3)	2.975 (2)	110 (2)

Symmetry code: (i) 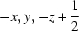.

**Fig. 1.**
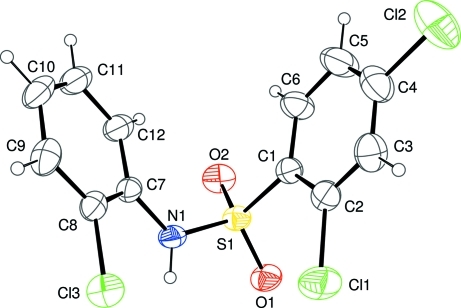
Molecular structure of (I), showing the atom labelling scheme and displacement ellipsoids are drawn at the 50% probability level.

**Fig. 2.**
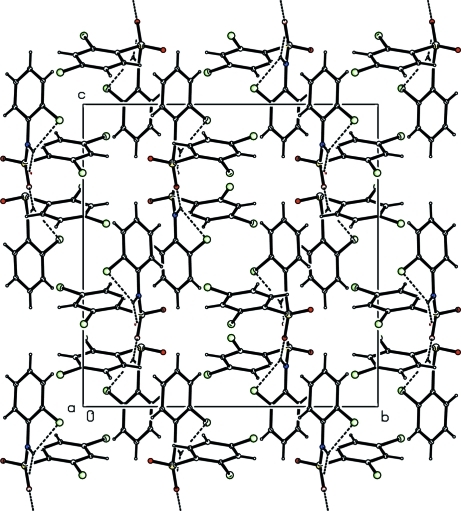
Molecular packing of (I) with hydrogen bonding shown as dashed lines.

## supplementary crystallographic information

### Comment

As a part of studying the substituent effects on the structures of *N*-(aryl)arylsulfonamides (Gowda *et al. *, 2010*a,b*; Nirmala *et al.*, 2010), the structure of 2,4-dichloro-*N*-(2-chlorophenyl)-benzenesulfonamide (I) has been determined (Fig. 1). The conformation of the N—H bond in the C—SO_2_—NH—C segment is *syn* to the *ortho*-Cl in the aniline ring (Fig. 1), contrary to the *anti* conformation observed between the N—H bond and *meta*-Cl in the aniline ring of 2,4-dichloro-*N*-(3-chlorophenyl)-benzenesulfonamide (II) (Gowda *et al.*, 2010*a*).

The molecule is twisted at the S atom with the C1—SO_2_—NH—C7 torsion angle of 54.5 (3)°, compared to the values of 62.3 (2)° in (II), 67.8 (2)° in 2,4-dichloro-*N*-(4-chlorophenyl)benzenesulfonamide (III)(Nirmala *et al.*, 2010), 55.1 (3)° (molecule 1) and -48.3 (3)° (molecule 2) in 2,4-dichloro-*N*-(phenyl)-benzenesulfonamide (IV) (Gowda *et al.*, 2010*b*). The sulfonyl benzene and the aniline benzene rings in (I) are tilted relative to each other by 74.3 (1)°, compared to the values of 69.3 (1)° in (II), 65.0 (1)° in (III), 80.5 (2)° in the molecule 1 and 64.9 (1)° in molecule 2 of (IV).

The other bond parameters in (I) are similar to those observed in (II), (III), (IV) and other aryl sulfonamides (Perlovich *et al.*, 2006; Gelbrich *et al.*, 2007).

In the crystal structure, the pairs of intermolecular N–H···O hydrogen bonds (Table 1) link the molecules *via* dimers into infinite sequences running parallel to the *c*-axis. Part of the crystal structure is shown in Fig. 2.

### Experimental

The solution of 1,3-dichlorobenzene (10 cc) in chloroform (40 cc) was treated dropwise with chlorosulfonic acid (25 cc) at 0 ° C. After the initial evolution of hydrogen chloride subsided, the reaction mixture was brought to room temperature and poured into crushed ice in a beaker. The chloroform layer was separated, washed with cold water and allowed to evaporate slowly. The residual 2,4-dichlorobenzenesulfonylchloride was treated with 2-chloroaniline in the stoichiometric ratio and boiled for ten minutes. The reaction mixture was then cooled to room temperature and added to ice cold water (100 cc). The resultant solid 2,4-dichloro-*N*-(2-chlorophenyl)benzenesulfonamide was filtered under suction and washed thoroughly with cold water. It was then recrystallized to constant melting point from dilute ethanol. The purity of the compound was checked and characterized by recording its infrared and NMR spectra (Savitha & Gowda, 2006). Prism like colorless single crystals used in X-ray diffraction studies were grown in ethanolic solution by slow evaporation at room temperature.

### Refinement

The H atom of the NH group was located in a difference map and later restrained to N—H = 0.86 (1)Å. The other H atoms were positioned with idealized geometry using a riding model with C—H = 0.93 Å. All H atoms were refined with isotropic displacement parameters (set to 1.2 times of the *U*_eq_ of the parent atom).

### Crystal data

**Table:**

C_12_H_8_Cl_3_NO_2_S	*F*(000) = 1360
*M**_r_* = 336.60	*D*_x_ = 1.558 Mg m^−^^3^
Orthorhombic, *P**b**c**n*	Mo *K*α radiation, λ = 0.71073 Å
Hall symbol: -P 2n 2ab	Cell parameters from 2957 reflections
*a* = 10.5639 (8) Å	θ = 2.5–27.8°
*b* = 16.279 (1) Å	µ = 0.78 mm^−^^1^
*c* = 16.693 (1) Å	*T* = 299 K
*V* = 2870.7 (3) Å^3^	Prism, colourless
*Z* = 8	0.30 × 0.24 × 0.22 mm

### Data collection

**Table:**

Oxford Diffraction Xcalibur diffractometer with a Sapphire CCD detector	2938 independent reflections
Radiation source: fine-focus sealed tube	2054 reflections with *I* > 2σ(*I*)
graphite	*R*_int_ = 0.019
Rotation method data acquisition using ω and phi scans	θ_max_ = 26.4°, θ_min_ = 2.5°
Absorption correction: multi-scan (*CrysAlis RED*; Oxford Diffraction, 2009)	*h* = −9→13
*T*_min_ = 0.800, *T*_max_ = 0.847	*k* = −14→20
8054 measured reflections	*l* = −11→20

### Refinement

**Table:**

Refinement on *F*^2^	Primary atom site location: structure-invariant direct methods
Least-squares matrix: full	Secondary atom site location: difference Fourier map
*R*[*F*^2^ > 2σ(*F*^2^)] = 0.042	Hydrogen site location: inferred from neighbouring sites
*wR*(*F*^2^) = 0.107	H atoms treated by a mixture of independent and constrained refinement
*S* = 1.01	*w* = 1/[σ^2^(*F*_o_^2^) + (0.0458*P*)^2^ + 1.5152*P*] where *P* = (*F*_o_^2^ + 2*F*_c_^2^)/3
2938 reflections	(Δ/σ)_max_ < 0.001
175 parameters	Δρ_max_ = 0.36 e Å^−^^3^
1 restraint	Δρ_min_ = −0.35 e Å^−^^3^

### Special details

**Table:**

Experimental. CrysAlis RED (Oxford Diffraction, 2009) Empirical absorption correction using spherical harmonics, implemented in SCALE3 ABSPACK scaling algorithm.
Geometry. All e.s.d.'s (except the e.s.d. in the dihedral angle between two l.s. planes) are estimated using the full covariance matrix. The cell e.s.d.'s are taken into account individually in the estimation of e.s.d.'s in distances, angles and torsion angles; correlations between e.s.d.'s in cell parameters are only used when they are defined by crystal symmetry. An approximate (isotropic) treatment of cell e.s.d.'s is used for estimating e.s.d.'s involving l.s. planes.
Refinement. Refinement of *F*^2^ against ALL reflections. The weighted *R*-factor *wR* and goodness of fit *S* are based on *F*^2^, conventional *R*-factors *R* are based on *F*, with *F* set to zero for negative *F*^2^. The threshold expression of *F*^2^ > σ(*F*^2^) is used only for calculating *R*-factors(gt) *etc*. and is not relevant to the choice of reflections for refinement. *R*-factors based on *F*^2^ are statistically about twice as large as those based on *F*, and *R*- factors based on ALL data will be even larger.

### Fractional atomic coordinates and isotropic or equivalent isotropic displacement parameters (Å^2^)

**Table:**

	*x*	*y*	*z*	*U*_iso_*/*U*_eq_	
C1	0.3134 (2)	0.61639 (15)	0.32900 (15)	0.0497 (6)	
C2	0.2866 (3)	0.53340 (17)	0.31598 (16)	0.0579 (7)	
C3	0.3728 (3)	0.47403 (19)	0.3366 (2)	0.0753 (9)	
H3	0.3545	0.4188	0.3282	0.090*	
C4	0.4862 (3)	0.4967 (2)	0.3697 (2)	0.0850 (10)	
C5	0.5150 (3)	0.5775 (2)	0.3827 (2)	0.0919 (11)	
H5	0.5923	0.5920	0.4052	0.110*	
C6	0.4285 (3)	0.6371 (2)	0.36218 (19)	0.0713 (8)	
H6	0.4480	0.6921	0.3708	0.086*	
C7	0.0989 (2)	0.68618 (15)	0.45102 (14)	0.0477 (6)	
C8	0.0161 (2)	0.63852 (15)	0.49537 (15)	0.0509 (6)	
C9	0.0238 (3)	0.63637 (17)	0.57821 (16)	0.0664 (8)	
H9	−0.0334	0.6050	0.6076	0.080*	
C10	0.1164 (3)	0.68083 (19)	0.61698 (17)	0.0730 (9)	
H10	0.1232	0.6786	0.6725	0.088*	
C11	0.1978 (3)	0.7281 (2)	0.57371 (17)	0.0703 (8)	
H11	0.2600	0.7581	0.6000	0.084*	
C12	0.1892 (3)	0.73195 (18)	0.49182 (16)	0.0629 (7)	
H12	0.2444	0.7655	0.4633	0.075*	
N1	0.08883 (19)	0.68827 (15)	0.36630 (12)	0.0542 (6)	
H1N	0.0212 (16)	0.6693 (15)	0.3452 (15)	0.065*	
O1	0.15449 (18)	0.68354 (12)	0.22707 (9)	0.0648 (5)	
O2	0.27286 (18)	0.77149 (11)	0.32041 (12)	0.0675 (5)	
Cl1	0.14598 (8)	0.50245 (5)	0.27304 (6)	0.0914 (3)	
Cl2	0.59587 (12)	0.42218 (8)	0.39456 (10)	0.1453 (5)	
Cl3	−0.09873 (7)	0.58020 (5)	0.44734 (5)	0.0691 (2)	
S1	0.20683 (6)	0.69660 (4)	0.30490 (4)	0.05119 (19)	

### Atomic displacement parameters (Å^2^)

**Table:**

	*U*^11^	*U*^22^	*U*^33^	*U*^12^	*U*^13^	*U*^23^
C1	0.0463 (14)	0.0562 (15)	0.0466 (14)	−0.0060 (12)	0.0030 (11)	0.0018 (12)
C2	0.0561 (16)	0.0593 (16)	0.0584 (16)	−0.0085 (14)	0.0062 (13)	−0.0016 (13)
C3	0.082 (2)	0.0563 (17)	0.087 (2)	0.0018 (17)	0.0103 (18)	0.0005 (17)
C4	0.075 (2)	0.083 (2)	0.097 (3)	0.024 (2)	−0.0010 (19)	0.006 (2)
C5	0.0572 (19)	0.099 (3)	0.119 (3)	0.0074 (19)	−0.0242 (18)	−0.003 (2)
C6	0.0537 (17)	0.0678 (19)	0.092 (2)	−0.0065 (15)	−0.0125 (16)	−0.0029 (17)
C7	0.0517 (14)	0.0510 (14)	0.0403 (13)	0.0108 (12)	−0.0058 (11)	−0.0019 (11)
C8	0.0630 (16)	0.0433 (13)	0.0463 (14)	0.0131 (12)	−0.0027 (12)	0.0000 (12)
C9	0.093 (2)	0.0583 (17)	0.0482 (16)	0.0183 (17)	0.0063 (15)	0.0079 (14)
C10	0.110 (3)	0.069 (2)	0.0401 (15)	0.0277 (19)	−0.0159 (16)	−0.0087 (15)
C11	0.086 (2)	0.0712 (19)	0.0535 (17)	0.0103 (17)	−0.0189 (15)	−0.0123 (15)
C12	0.0659 (17)	0.0709 (19)	0.0518 (16)	−0.0033 (15)	−0.0088 (13)	−0.0053 (14)
N1	0.0456 (12)	0.0765 (16)	0.0404 (11)	−0.0022 (11)	−0.0071 (9)	−0.0013 (11)
O1	0.0674 (11)	0.0888 (14)	0.0382 (9)	−0.0078 (11)	−0.0027 (8)	0.0105 (9)
O2	0.0745 (13)	0.0518 (10)	0.0763 (13)	−0.0140 (10)	−0.0064 (10)	0.0104 (10)
Cl1	0.0830 (6)	0.0772 (5)	0.1140 (7)	−0.0306 (5)	−0.0171 (5)	−0.0062 (5)
Cl2	0.1218 (9)	0.1269 (10)	0.1873 (13)	0.0642 (8)	−0.0140 (9)	0.0199 (9)
Cl3	0.0743 (5)	0.0665 (5)	0.0665 (5)	−0.0100 (4)	−0.0044 (4)	0.0089 (4)
S1	0.0528 (4)	0.0574 (4)	0.0434 (3)	−0.0060 (3)	−0.0025 (3)	0.0086 (3)

### Geometric parameters (Å, °)

**Table:**

C1—C6	1.379 (3)	C7—N1	1.419 (3)
C1—C2	1.397 (3)	C8—C9	1.386 (4)
C1—S1	1.770 (3)	C8—Cl3	1.737 (3)
C2—C3	1.372 (4)	C9—C10	1.378 (4)
C2—Cl1	1.725 (3)	C9—H9	0.9300
C3—C4	1.369 (5)	C10—C11	1.361 (4)
C3—H3	0.9300	C10—H10	0.9300
C4—C5	1.367 (5)	C11—C12	1.372 (4)
C4—Cl2	1.728 (3)	C11—H11	0.9300
C5—C6	1.377 (4)	C12—H12	0.9300
C5—H5	0.9300	N1—S1	1.620 (2)
C6—H6	0.9300	N1—H1N	0.855 (10)
C7—C8	1.383 (4)	O1—S1	1.4279 (17)
C7—C12	1.389 (3)	O2—S1	1.4282 (18)
			
C6—C1—C2	118.5 (3)	C9—C8—Cl3	119.2 (2)
C6—C1—S1	118.1 (2)	C10—C9—C8	119.8 (3)
C2—C1—S1	123.3 (2)	C10—C9—H9	120.1
C3—C2—C1	120.5 (3)	C8—C9—H9	120.1
C3—C2—Cl1	118.1 (2)	C11—C10—C9	119.7 (3)
C1—C2—Cl1	121.4 (2)	C11—C10—H10	120.1
C4—C3—C2	119.5 (3)	C9—C10—H10	120.1
C4—C3—H3	120.3	C10—C11—C12	120.9 (3)
C2—C3—H3	120.3	C10—C11—H11	119.6
C5—C4—C3	121.2 (3)	C12—C11—H11	119.6
C5—C4—Cl2	119.2 (3)	C11—C12—C7	120.6 (3)
C3—C4—Cl2	119.6 (3)	C11—C12—H12	119.7
C4—C5—C6	119.5 (3)	C7—C12—H12	119.7
C4—C5—H5	120.3	C7—N1—S1	125.13 (17)
C6—C5—H5	120.3	C7—N1—H1N	117.7 (19)
C5—C6—C1	120.8 (3)	S1—N1—H1N	114.3 (19)
C5—C6—H6	119.6	O1—S1—O2	118.76 (12)
C1—C6—H6	119.6	O1—S1—N1	105.39 (11)
C8—C7—C12	118.2 (2)	O2—S1—N1	109.43 (12)
C8—C7—N1	120.0 (2)	O1—S1—C1	110.08 (12)
C12—C7—N1	121.8 (2)	O2—S1—C1	106.14 (12)
C7—C8—C9	120.7 (3)	N1—S1—C1	106.48 (12)
C7—C8—Cl3	120.06 (19)		
			
C6—C1—C2—C3	0.7 (4)	Cl3—C8—C9—C10	178.5 (2)
S1—C1—C2—C3	−179.4 (2)	C8—C9—C10—C11	1.5 (4)
C6—C1—C2—Cl1	−178.8 (2)	C9—C10—C11—C12	−0.2 (5)
S1—C1—C2—Cl1	1.1 (3)	C10—C11—C12—C7	−1.5 (4)
C1—C2—C3—C4	−0.4 (5)	C8—C7—C12—C11	1.7 (4)
Cl1—C2—C3—C4	179.0 (3)	N1—C7—C12—C11	−179.2 (3)
C2—C3—C4—C5	0.1 (5)	C8—C7—N1—S1	−145.6 (2)
C2—C3—C4—Cl2	−179.1 (2)	C12—C7—N1—S1	35.3 (4)
C3—C4—C5—C6	0.0 (6)	C7—N1—S1—O1	171.4 (2)
Cl2—C4—C5—C6	179.3 (3)	C7—N1—S1—O2	−59.8 (2)
C4—C5—C6—C1	0.2 (5)	C7—N1—S1—C1	54.5 (2)
C2—C1—C6—C5	−0.5 (4)	C6—C1—S1—O1	134.6 (2)
S1—C1—C6—C5	179.6 (3)	C2—C1—S1—O1	−45.3 (2)
C12—C7—C8—C9	−0.3 (4)	C6—C1—S1—O2	4.9 (2)
N1—C7—C8—C9	−179.4 (2)	C2—C1—S1—O2	−175.0 (2)
C12—C7—C8—Cl3	179.94 (19)	C6—C1—S1—N1	−111.6 (2)
N1—C7—C8—Cl3	0.8 (3)	C2—C1—S1—N1	68.5 (2)
C7—C8—C9—C10	−1.3 (4)		

### Hydrogen-bond geometry (Å, °)

**Table:**

*D*—H···*A*	*D*—H	H···*A*	*D*···*A*	*D*—H···*A*
N1—H1N···O1^i^	0.86 (1)	2.23 (2)	3.007 (3)	152 (2)
N1—H1N···Cl3	0.86 (1)	2.57 (3)	2.975 (2)	110 (2)

Symmetry codes: (i) −*x*, *y*, −*z*+1/2.
